# Freeze–thaw cycle frequency affects root growth of alpine meadow through changing soil moisture and nutrients

**DOI:** 10.1038/s41598-022-08500-w

**Published:** 2022-03-15

**Authors:** Zihao Man, Changkun Xie, Ruiyuan Jiang, Shengquan Che

**Affiliations:** School of Design, Shanghai Jiao Tong University, Shanghai, 200240 China

**Keywords:** Ecology, Hydrology

## Abstract

Alpine meadows grow in alpine regions and play an important role in the production and life of alpine regions. As a unique feature of alpine regions, freeze–thaw cycles (FTCs) affect the growth of alpine meadows. However, with climate change, the change in the freeze–thaw cycle frequency (FTCF) has become obvious. These changes affect the content and distribution of soil moisture and nutrients, as well as the growth of roots in the alpine meadow. Therefore, based on the analysis of FTCF in the Nagqu River Basin, the characteristics of soil moisture, nutrients, and alpine meadow roots are analyzed, thus revealing the influence mechanism of FTCF on the root growth of alpine meadows. The results highlight three major findings. (1) Compared with the low-frequency mode (LFM), the moisture at 0–20, 20–40, and 40–60 cm in the high-frequency mode (HFM) has decreased by 30.74%, 52.89%, and 47.52%, respectively. Additionally, in HFM the contents of soil hydrolysable nitrogen (HN), available K (AK), and microbial biomass carbon (MBC) at the same depth are lower than those in LFM. (2) The original distribution of soil moisture at 0–60 cm has gradually increased from the surface to the bottom. However, with the increase in FTCF, the distribution of the soil moisture now means that the soil moisture at the surface (0–20 cm) and the deeper layers (40–60 cm) is higher than that in the middle (20–40 cm). (3) With the increase in FTCF, the growth mode of alpine meadow roots has changed from vertical extension to horizontal divergence; the distribution range of roots has changed from 0–40 cm to 0–20 cm; the length, surface area, and volume of 0–0.5 mm roots have increased by 20.95 cm, 1.90 cm^2^, and 0.014 cm^3^; and the corresponding specific gravity has increased by 9.09%, 13.50%, and 12.14%, respectively. This study provides a theoretical basis for predicting the growth mode of alpine meadow roots in the Nagqu River Basin under the influence of climate change and provides guidance for protecting the ecology of alpine regions and mitigating and solving global climate change.

## Introduction

As an important carbon source on land, soil ecosystems make up about 20% of the global carbon store^1^, with meadows making a huge contribution. The frozen soil ecosystem in alpines region is a very unique land ecosystem in which unique alpine meadows grow^2–4^. Alpine meadows play a vital role in water and soil conservation, soil restoration, animal husbandry development, and ecosystem functions and services in alpine regions^5–9^, but alpine meadows are extremely sensitive to environmental changes and thus extremely fragile^10–13^.

The Qinghai–Tibet Plateau is the youngest and highest plateau in the world^14^. It is a natural experimental area for studying the evolution of life and the relationship between humans and land in alpine regions^15^. Seasonal frozen soil interacts with the atmosphere through dynamic changes in water and heat in the active layer. The freeze–thaw cycle (FTC) is one of the most significant physical characteristics of the alpine permafrost region^2,16^. The freeze–thaw cycle frequency (FTCF) describes the average number of FTCs per day during the period when FTCs occur, it has a significant impact on the growth and survival of alpine meadows.

FTC can directly affect the growth of alpine meadows. Low temperature reduces the activity and the metabolism rate of root cells, and slows down the absorption rate of nutrients^17–19^, even leads to the phase transition of alpine meadow root cell membrane and destroys the cell structure^20^.At the same time, anaerobic respiration occurs in roots, and the secretion accumulation produced by anaerobic respiration also reduces its physiological activity, which ultimately inhibits the growth of alpine meadow. Frequent low-temperature disturbances have more effects on the adaptability of alpine meadow roots, further damaging the alpine meadow root cells. In addition, FTC can also indirectly affect the growth of alpine meadows. Water volume expands when moisture in the soil is frozen and shrinks when it melts. These changes can cause the disintegration of soil aggregates; change the physical and chemical properties of soil, such as particle diameter, porosity, and organic matter^21–29^; and then reflect the storage and migration of soil moisture and nutrients^28^. In recent years, with global warming^30^, the carbohydrates stored in frozen soil are gradually released into the atmosphere, which further affects local and even global climate change. However, a series of environmental changes, such as temperature rises and the frequent occurrence of abnormal weather, have had a great impact on the soil moisture and temperature. The original freeze–thaw cycle frequency (FTCF) of the frozen soil ecosystem on the Qinghai–Tibet Plateau is bound to change^28,31–34^, and the soil properties may change irreversibly, ultimately affecting the growth mode of alpine meadow roots.

However, due to the sensitivity and vulnerability of the frozen soil ecosystem^22^, all effects may be amplified and the succession process of alpine meadow ecosystems may be shortened^28^, leading to a series of unforeseen hazards. Recent studies have focused on the relationship between vegetation and low temperatures or FTCs. However, studies on the effect mechanism of FTCF on alpine meadows are scarce, and research on the mechanism of FTCF on alpine meadow root distribution is even rarer.

In this study, we measured the morphological structure of alpine meadow roots and the contents of water and nutrients at different depths of soil. We hypothesized that, due to climate change, the FTCF will gradually increase. Frequent FTC will destroy soil structure, change the original physical and chemical properties of soil, and lead to changes in the content and distribution of water and nutrients. Alpine meadows will adapt to environmental changes by adjusting growth mode of alpine meadow roots. The aims of this study are (1) to analyze the influence of FTCF on the content and distribution of soil moisture and nutrients; (2) to analyze the influence of FTCF on the distribution range, type and density of alpine meadow roots; (3) to reveal the influence mechanism of FTCF on the growth of alpine meadow roots; and (4) to explore the growth mode of alpine meadow roots in the Nagqu River Basin under the effects of climate change.

## Materials and methods

### Study area

**Figure 1:**
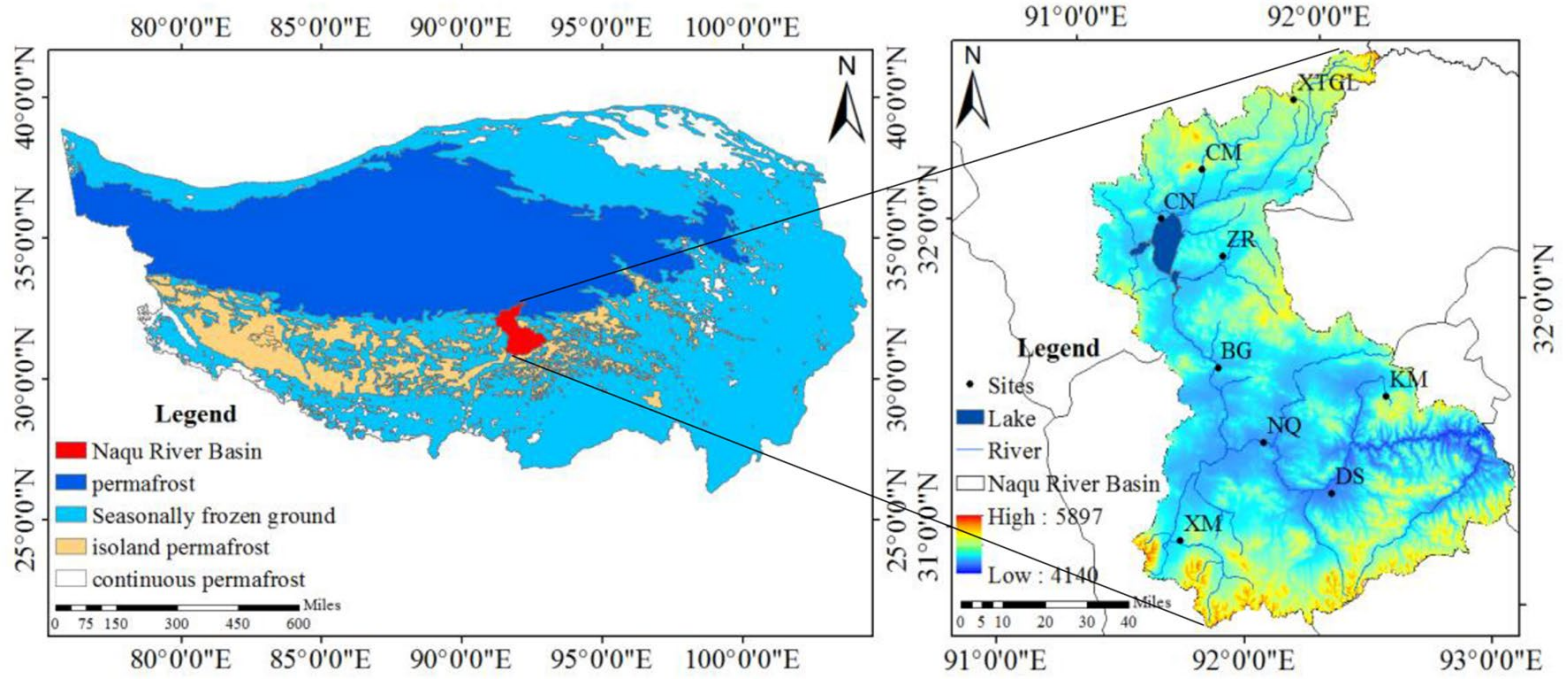
Location map of the Nagqu River Basin. The different colors in the (left) figure indicate different types of frozen soil, and the different colors in the (right) figure indicate different altitudes, XM is the sampling point in Xiangmao Town, CM is the sampling point in Cuoma Town. The figure is generated with use of ArcGis 10.8 (https://www.esri.com/en-us/home).

The Nagqu River Basin is located Tanggula, Nyainqentanglha, and Gangdise, and has an area of 16,328 km^2^. In the Nagqu River Basin, the average elevation is greater than 4500 m, the annual average temperature is − 0.6 ℃, the annual average precipitation is about 477.8 mm, the annual average wind speed is about 2.5 m/s, and the annual average sunshine hours are 2723 h. The main types of frozen soil in the Nagqu River Basin are permafrost and seasonal frozen soil, as shown in Fig. 1. Most areas of the Nagqu River Basin have felty soils, covering an area of 11,018.3 km^2^ and accounting for 67.5% of the basin area. The main vegetation in the Nagqu River Basin includes *Kobresia bellardii*, *Kobresia humilis*, and *Kobresia parva*.

Altitude and vegetation coverage are the most important factors affecting soil water and heat changes, as well as the main influencing factors for differences in FTC. Therefore, we investigated 9 areas in the Nagqu River Basin and selected two experimental sites in Cuoma Town and Xiangmao Town according to the FTCF, altitude, and vegetation coverage. The altitude, vegetation coverage, and soil properties of these two experimental sites are similar, but the FTCF is very different, mainly due to the climate. The purpose of this choice is to control the environmental factors of the natural experimental site to be similar, except for the FTCF. The soil properties are shown in Table 1.

**Table 1:** Soil property information at different sites.

Sites	Altitude (m)	Vegetation coverage (%)	Soil bulk density (g/cm^3^)	Soil porosity (%)
CM	4760	70	1.26	79.13
XM	4730	60	1.29	71.77

### Data collection and processing

#### Meteorological data

The instrument used for determining the soil temperature and volumetric water content (VWC) is the SM300 soil sensor from Delta, UK, which has an accuracy of ± 0.1 °C and a ± 0.1% VWC. The sensor was buried in 5 cm of soil at Cuoma (CM), Xiangmao (XM). Data concerning the top soil (5–10 cm) temperature and moisture from 1 January to 30 June were obtained in 2018 (recorded every 30 min).

#### Alpine meadow roots

In May 2018, we buried five transparent root canals in XM and CM. The microhabitats around the root canals were similar and the vegetation types were uniform (The alpine meadow in this study is mainly *Kobresia pygmaea (C. B. Clarke)*). From 11 to 14 September 2018, we measured the root distribution characteristics of alpine meadows with a mini-rhizotron instrument.

The shooting range of the mini-rhizotron instrument lens is 1.35 × 1.35 cm. We first photographed the root on the ground surface (the depth is 0 cm). In order to ensure the integrity of the roots in the same layer, after each photo was taken we rotated it 45° to continue shooting, recording eight photos for each layer. After shooting one layer, we moved down one layer at a distance of 1.35 cm and continued shooting. The shooting ended when it became difficult to find the roots in the lens of the mini-rhizotron instrument.

#### Soil nutrient and moisture data

In September 2018, soil samples of the 0–20, 20–40, and 40–60 cm soil layers were collected by five-point mixed sampling method (Five parts of soil are evenly mixed into one part), each group had a set of 3 replicates. The soil moisture was analyzed gravimetrically after drying the soil at 105 ℃ for 24 h; the soil available phosphorus (AP) was analyzed using the Olsen method^35^; the soil hydrolysable nitrogen (HN) was analyzed using the alkaline lysis diffusion method^36^; and the soil available K (AK) was analyzed by filtering a soil solution containing 50 mL of CH_3_COOHN_4_ (1 mol/L) and using a spectrophotometer to measure the filtrate. After the soil was fumigated with chloroform, the carbon content in the extract was determined, and the soil microbial biomass carbon (MBC) was calculated by the difference between the carbon content in the fumigated and non-fumigated soil.

### Freeze–thaw cycle frequency index

In ecosystems, FTC affects the physical and chemical properties of soil and the growth of vegetation and microorganisms^18,25^. Although the freeze–thaw cycle days (FTCD) and the number of freeze–thaw cycles (NFTC) can reflect the freeze–thaw characteristics of soil, the freeze–thaw cycle frequency (FTCF) index can better measure the impact of repeated FTCs on alpine meadow roots. This view has been proven by Man et al^28^. It can be expressed by:

1 $$ {\text{FTCF = }}\frac{{{\text{NFTC}}}}{{{\text{FTCD}}}} $$

where FTCD is the number of freeze–thaw cycle days, referring to how many days in a year the FTC occurs; NFTC is the number of freeze–thaw cycles, referring to how many FTCs occur during the period when the FTC occurs; and FTCF is the freeze–thaw cycle frequency, referring to the average number of FTCs per day during the period when the FTC occurs.

Using the above formula for the calculations, the FTC characteristics of XM and CM are shown in the following Table 2.

**Table 2:** Freeze–thaw cycle characteristics of surface soil at CM and XM.

Monitoring sites	Starting time	Closing time	NFTC	FTCD	FTCF
XM	9 January 2018	28 March 2018	80	78	1.02
CM	8 February 2018	8 March 2018	12	28	0.43

### Statistical analysis

The Corrplot program in R language and the Origin was used to analyze the correlation between FTCF and soil properties.

### Declarations

All experiments on plants adherence to relevant ethical parameters. All experiments are approved by regional authorities. The alpine meadow in this study is mainly Kobresia pygmaea (C. B. Clarke). Zihao Man undertook the formal identification of the plant material used in the study, and the pictures were preserved. And Xiangmao Township, Dasa Township, Kongma Township, Zharen Township, and Cuoma Township government all approved soil collection.

## Results

### Morphological distribution of alpine meadow roots

**Figure 2:**
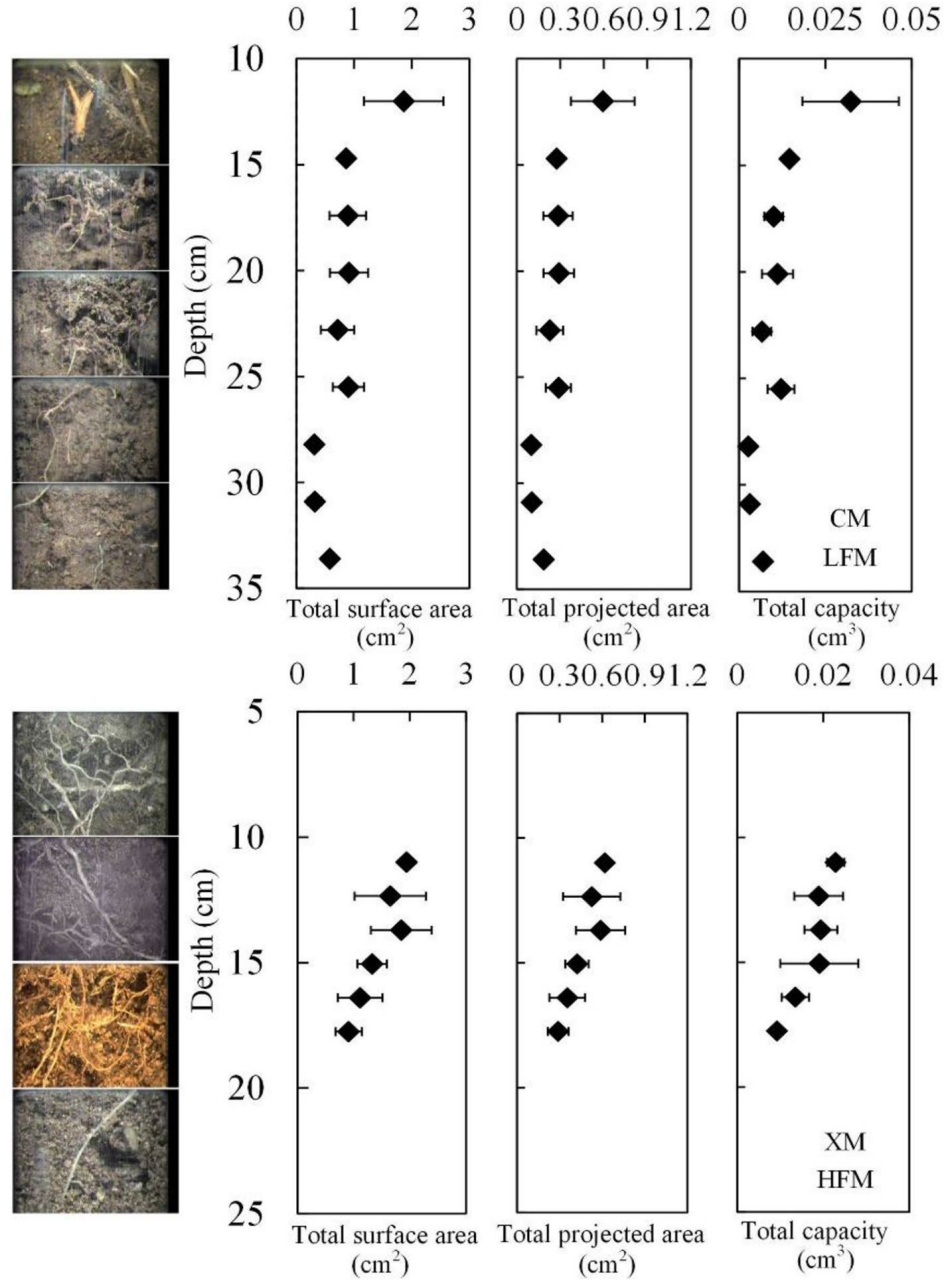
Distribution of alpine meadow roots at CM and XM. The figure is generated with use of Excel 2019 (https://www.microsoft.com/zh-cn/microsoft-365/excel).

As shown in Fig. 2, in XM, the alpine meadow roots are mainly distributed at the level of 0–20 cm. The distribution of alpine meadow roots is wider than that of CM, which is mainly distributed from 0 to 40 cm. The alpine meadow roots in XM have a larger surface area, a larger projected area, and a larger volume than that in CM. In XM, the distribution law of alpine meadow roots is horizontal divergence, while the distribution of alpine meadow roots in CM shows vertical extension, but the alpine meadow roots all decrease gradually from the top to the bottom in the XM and CM. At the same soil depth, the total length, total surface area, total projected area, and total volume of alpine meadow roots in XM are larger than those in CM.

**Figure 3:**
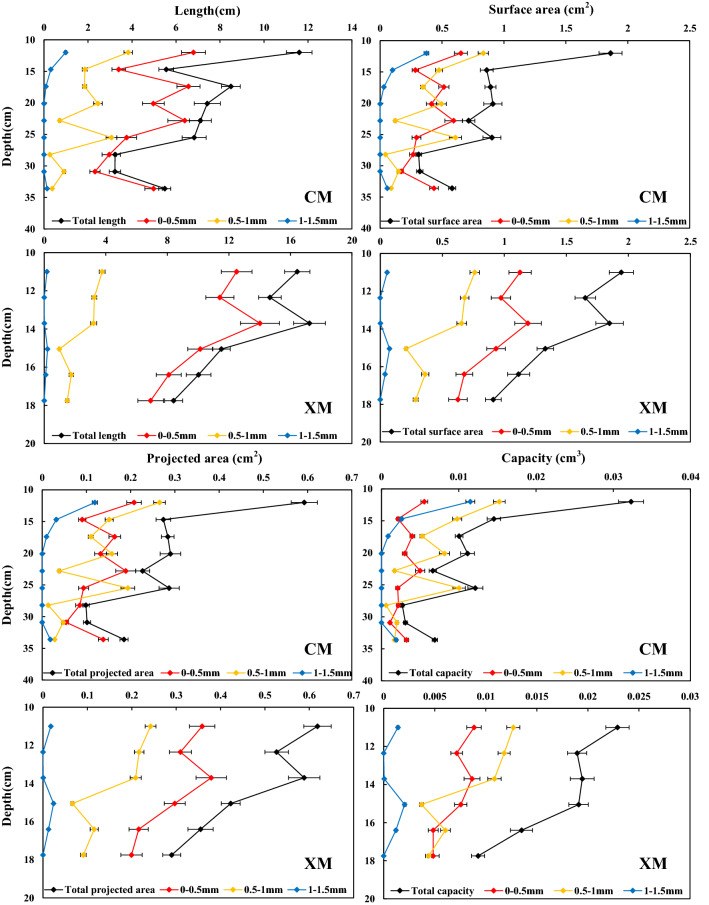
Distribution of root length, surface area, projected area and volume. The figure is generated with use of Excel 2019 (https://www.microsoft.com/zh-cn/microsoft-365/excel).

**Figure 4:**
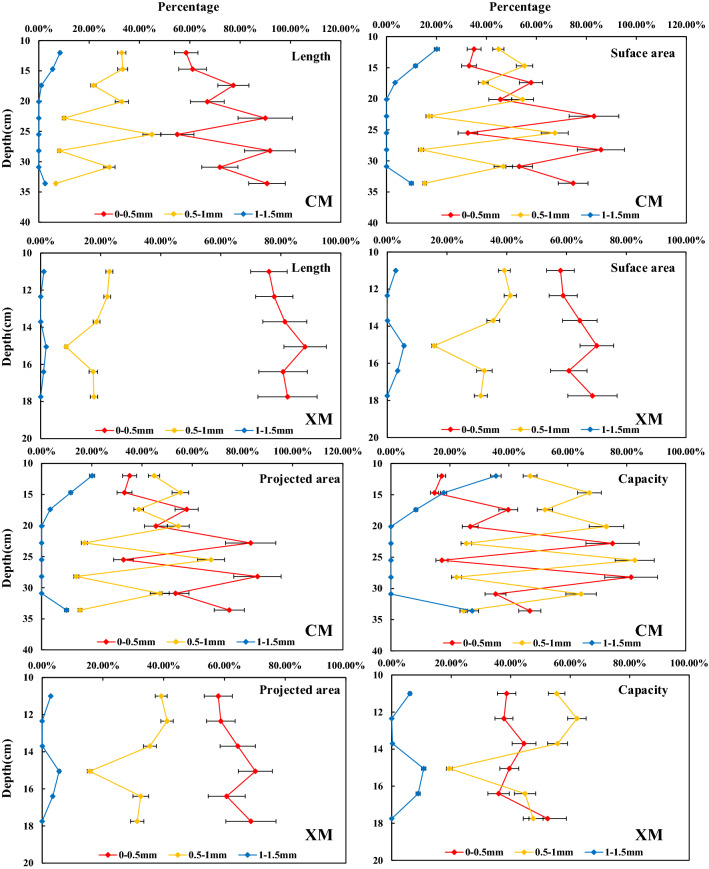
Distribution and ratio of root length, surface area, projected area and volume. The figure is generated with use of Excel 2019 (https://www.microsoft.com/zh-cn/microsoft-365/excel).

According to the diameter of the roots, the alpine meadow roots in the Nagqu River Basin can be divided into three types: 0–0.5, 0.5–1, and 1–1.5 mm (Figs. 3 and 4). In CM and XM the length of the alpine meadow roots decreases with the increase in the soil depth. Additionally, at a soil depth of 0–60 cm, roots with a length of 0–0.5 mm account for the largest proportion, while roots with a length of 1–1.5 mm account for the smallest proportion. With the increase in soil depth, the proportion of the roots with lengths of 0.5–1 and 1–1.5 mm roots gradually decreases or even disappears, while the proportion with a length of 0–0.5 mm gradually increases.

As shown in Figs. 3 and 4, the surface area and the projected area of the alpine meadow roots in the Nagqu River Basin experience similar changes with depth. In CM, with the increase in soil depth, the proportion of roots with a surface area of 0–0.5 mm gradually increases and the proportion of roots with a surface area of 0.5–1 and 1–1.5 mm gradually decreases. In the 10–15 cm soil, roots with a surface area of 0.5–1 mm account for the largest proportion in CM; meanwhile, in XM the roots with a surface area of 0–0.5 mm account for the largest proportion in the 10–20 cm soil.

In CM, the roots with a volume of 0.5–1 mm roots account for the largest proportion in the 10–20 cm soil. However, the roots with a volume of 0.5–1 mm account for the largest proportion in the 10–15 cm soil, while the roots with a volume of 0–0.5 mm account for the largest proportion in the 15–20 cm soil.

In short, with the increase in soil depth, the length, surface area, projected area, and volume of the alpine meadow roots gradually decrease in the Nagqu River Basin, the proportion of 0–0.5 mm roots increases while the proportion of 0.5–1 mm roots decreases. The distribution of alpine meadow roots in CM shows a vertical extension, while in XM it shows horizontal divergence. In addition, compared with CM, the total length, total surface area, and total volume of 0–0.5 mm roots in XM increase by 20.95 cm, 1.90 cm^2^, and 0.014 cm^3^, and the corresponding specific gravity increases by 9.09%, 13.50%, and 12.14%. The total length, total surface area, and total volume of 0.5–1 and 1–1.5 mm roots in XM show smaller changes, and the corresponding specific gravity decreases.

### Distribution of soil moisture and nutrients

**Figure 5:**
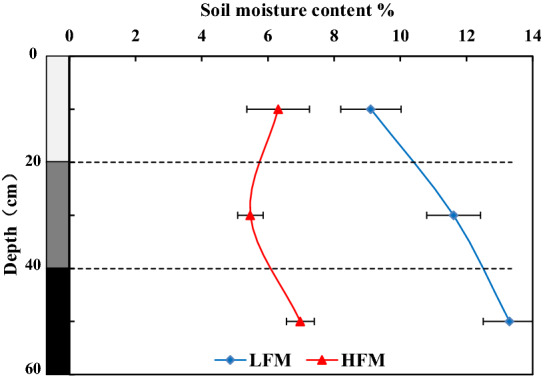
Soil moisture distribution in LFM and HFM. The figure is generated with use of Excel 2019 (https://www.microsoft.com/zh-cn/microsoft-365/excel).

As shown in Fig. 5, at the same soil depth, compared with LFM, the moisture in the 0–20, 20–40, and 40–60 cm soil in HFM was reduced by 30.74%, 52.89%, and 47.52%, and even the maximum soil moisture in the HFM was lower than that in the minimum soil moisture in LFM.

In LFM, as the soil depth increases, the soil moisture gradually increases. The 10–20 cm soil had the lowest moisture content in LFM of around 9.11%, the 20–40 cm soil moisture increased by 27.4%, and the 40–60 cm soil moisture increased by 45.9%. In HFM, the moisture content was 6.31% in the 0–20 cm soil. The moisture was the highest in the 40–60 cm soil. Compared with the 0–20 cm, the moisture was increased by 10.6%. The moisture was the lowest in the 20–40 cm soil, being reduced by 13.3% compared to the 0–20 cm soil. Therefore, HFM and LFM have different soil moisture distributions. In the 0–60 cm soil layer of HFM, the middle soil (20–40 cm) had a lower moisture content, while the surface (0–20 cm) and deep soil layers (40–60 cm) had higher moisture contents.

**Figure 6:**
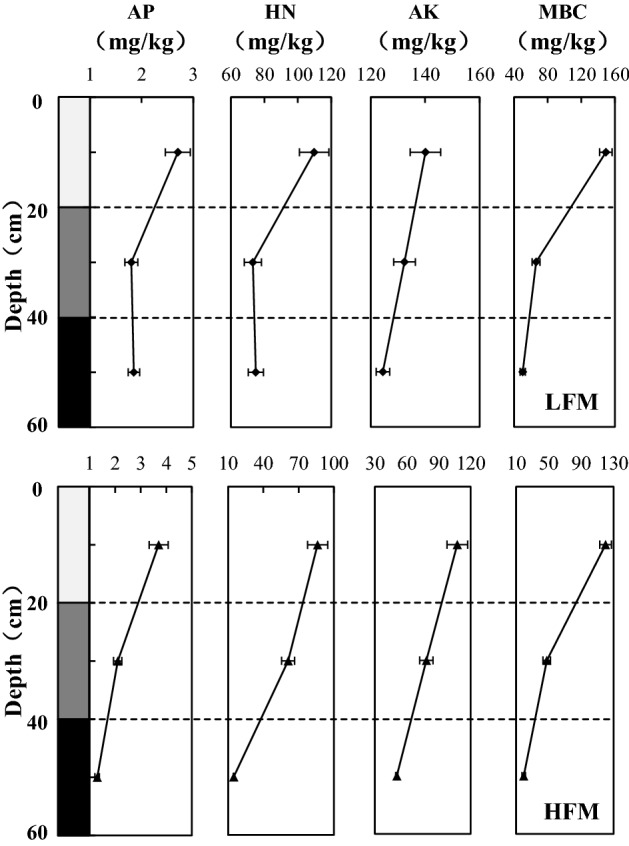
Soil nutrients distribution in LFM and HFM, HN is hydrolysable nitrogen, AP is available phosphorus, AK is available K, MBC is microbial biomass carbon. The figure is generated with use of Excel 2019 (https://www.microsoft.com/zh-cn/microsoft-365/excel).

In contrast to the distribution of soil moisture, the distribution of soil nutrients in HFM and LFM was the same: the soil nutrients gradually decreased from the surface to the bottom (Fig. 6). In LFM, the 0–20 cm-depth soil had the highest nutrient content, and the available phosphorous (AP), hydrolysable nitrogen (HN), available K (AK), and microbial biomass carbon (MBC) contents were 2.7, 109.83, 140.11, and 149.38 mg/kg, respectively. With the increase in soil depth, compared with the 0–20 cm soil, the contents of AP, HN, AK, and MBC in the 20–40 cm-depth soil were reduced by 33.33%, 33.44%, 5.45%, and 55.64%, while the content of AP, HN, AK, and MBC in the 40–60 cm-depth soil were reduced by 31.48%, 31.83%, 11.13%, and 66.28%, respectively. In HFM, the nutrient content in the 0–20 cm soil layer was also the largest, and the AP, HN, AK, and MBC has contents of 3.7, 86.17, 107.42, and 120.11 mg/kg, respectively. Compared with the 0–20 cm soil, the contents of AP, HN, AK, and MBC in the 20–40 cm-depth soil decreased by 43.24%, 29.11%, 27.07%, and 60.26%, respectively, while the contents of AP, HN, AK, and MBC in the 40–60 cm-depth soil decreased by 64.86%, 82.79%, 53.04%, and 83.88%, respectively.

Meanwhile, in the same depth of soil, in LFM the content of HN, AK, and MBC is greater than that in HFM. Compared with the LFM, the contents of HN, AK, and MBC in the 0–20 cm soil layer in HFM are reduced by 21.54%, 23.33%, and 19.59%; the HN, AK, and MBC in the 20–40 cm are decreased by 16.43%, 40.86%, and 27.98%; and the HN, AK, and MBC in the 40–60 cm decreased by 80.19%, 59.49%, 61.56%, respectively. However, the AP in the 0–20 and 20–40 cm depths in the HFM is greater than in the LFM. It may be that the higher FTCF causes more damage to Bradyrhizobium, Mesorhizobium, and Pseudomonas in the soil of the Nagqu River Basin, the competitiveness of Bacillus decreases and the abundance increases, while the phosphate-dissolving ability of Bacillus may lead to an increase in the phosphorus content in the soil^26^.

### Correlation analysis

**Figure 7:**
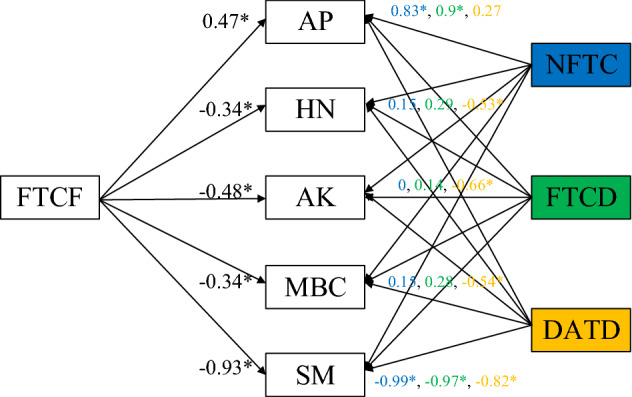
Correlation between FTCF and top soil moisture and nutrients content, HN is hydrolysable nitrogen, AP is available phosphorus, AK is available K, MBC is microbial biomass carbon, SM is soil moisture, FTCF is the freeze–thaw cycle frequency, FTCD is the number of freezing–thawing cycle days, NFTC is the number of freezing–thawing cycles, DATD is the daily average temperature difference, and *indicate the correlation coefficient is statistically significant at the *P* = 0.05 level. The figure is generated with use of R language 3.6.3 (https://www.r-project.org/) and Visio 2019 (https://www.microsoft.com/zh-cn/microsoft-365/visio/flowchart-software).

As shown in Fig. 7, the NFTC, FTCD, FTCF, and daily average temperature difference (DATD) all have a significant negative correlation with the top soil moisture in the Nagqu River Basin. This shows that the top soil moisture is not just affected by repeated FTC. Additionally, the effect of FTC on the top soil moisture is not transient; the longer FTC exists, the greater the impact on the top soil moisture will be.

Meanwhile, the contents of HN, AK, and MBC in the top soil have no obvious correlation with NFTC and FTCD but have a significant negative correlation with FTCF. This shows that, compared with NFTC and FTCD, FTCF is more suitable for measuring the influence of FTC characteristics on soil properties. With the increase in FTCF, the damage to the soil structure increases and the contents of HN, AK, and MBC in the top soil significantly decrease, but the AP shows different changes under the influence of microorganisms^28^.

## Discussion

### Effect of freeze–thaw cycle frequency on the distribution of soil nutrients and moisture

In LFM, the top soil (0–20 cm) contains the most nutrients (Fig. 6), because the top soil contains more organic matter, such as vegetation leaves, root litter, etc. These materials are an important source of top soil nutrients. With the increase in FTCF, the damage to the soil structure increases^28^ and the permeability of water increases^37^, which accelerated the vertical migration of nutrients. But the results of this study show (Fig. 6) that the nutrient content of top soil (0–20 cm) is also the highest in HFM, which indicates that although the loss process reduces the nutrient in the top soil, the nutrient loss is still less than the nutrient storage in the soil. And at the same soil depth, the nutrients in LFM are greater than in HFM (Fig. 6). This indicates that the larger the FTCF, the more soil nutrients are lost. In HFM, the greater the soil depth, the less nutrients, but in LFM, the difference in nutrient content between middle soil (20–40 cm) and deep soil (40–60 cm) is not significant (Fig. 6). This is because the larger the FTCF, the more damage to the soil structure and the more nutrient loss, but the resistance of the soil structure to FTC also increases as the soil depth increases. When the stability of soil structure can resist frequent FTC, the loss of soil nutrients no longer increases. Therefore, in the Nagqu River Basin, the nutrients affected by HFM may reach 40–60 cm soil, while the influence of LFM on soil nutrients does not exceed 40 cm.

As shown in Fig. 5, the content of soil water has increased from top to bottom in LFM. This is because the FTC in this study occurred during the thaw initiation period and the freeze initiation period^4^. During this period, the solid water in the soil gradually travels from top to bottom, and a large amount of liquid water gathers to the melting front, which leads to the migration of soil water from the top to the bottom^38,39^. However, the middle soil has less moisture than the top and deep soil in HFM (Fig. 5). The main reason is that during the thaw initiation period, the water in the top soil (0–20 cm) is easily replenished by rainfall, and this part of the replenishment accounts for a large proportion of top soil moisture. Therefore, there are two modes of soil moisture distribution in the Nagqu River Basin: In LFM, the soil moisture gradually increases from the top to bottom. In HFM, the top (0–20 cm) and deep layer (40–60 cm) has a higher moisture level, while the middle (20–40 cm) has a lower moisture content. In addition, the moisture in HFM is lower than in LFM (Fig. 5), which is determined by soil properties. With the increase in FTCF, the soil structure is destroyed, and the water-holding and water-conducting properties are changed, which is harmful to the preservation of soil water^28^, and a large amount of water moves downwards. Therefore, HFM can accelerate the transformation of soil water and groundwater.

### Effect of freeze–thaw cycle frequency on the distribution of alpine meadow roots

In LFM, alpine meadow roots are distributed in 0–40 cm soil, and as FTCF increases, the root distribution range is reduces to 0–20 cm soil (Fig. 2). This shows that the roots is plastic in morphology and physiology, and FTCM can affect the distribution of alpine meadow roots. Compared with the middle soil, the FTCF of top soil is greater, which is more harmful to the growth of alpine meadow roots^28^. But in HFM, alpine meadow roots choose to be distributed in the top soil rather than the deep soil (Fig. 2). This shows that although FTC can directly affect the growth of alpine meadow roots through low temperature and frequent temperature changes, it is the changes in soil moisture and nutrients that determine the distribution of alpine meadow roots. Of course, the content and distribution of soil moisture and nutrient are also affected by FTC.

For alpine meadows, different root diameters play different roles. Thinner roots are mainly used to absorb water and nutrients, while thicker roots are mainly used to transport these water and nutrients^40^. At the same soil depth, in HFM the proportion of length, surface area. and projected area of 0–0.5 mm roots are larger than those in LFM, and the length, surface area, and projected area of 0.5–1 mm roots are similar to those in LFM, but the proportion of the 0.5–1 mm roots length, surface area, and projected area are less than those in LFM (Figs. 3 and 4). This indicates that with the increase of FTCF, the ability of alpine meadow roots to transport water and nutrients is unchanged, but the ability to absorb water and nutrients is enhanced. The increase in FTCF not only increases the damage to roots, but also accelerates the loss of nutrients and impairs the ability of the soil to retain water^28^. Therefore, in order to absorb more water and nutrients, alpine meadows increase the proportion of 0–0.5 mm fine roots and the surface area of roots^41^. These results are consistent with the research results of Ma et al^42^. In a relatively harsh environment, the diameter range of plant roots is relatively narrow, which is an embodiment of adaptive behavior to adapt to the environment.

### The influence mechanism of freeze–thaw cycle frequency on the distribution of alpine meadow roots in Nagqu River Basin

**Figure 8:**
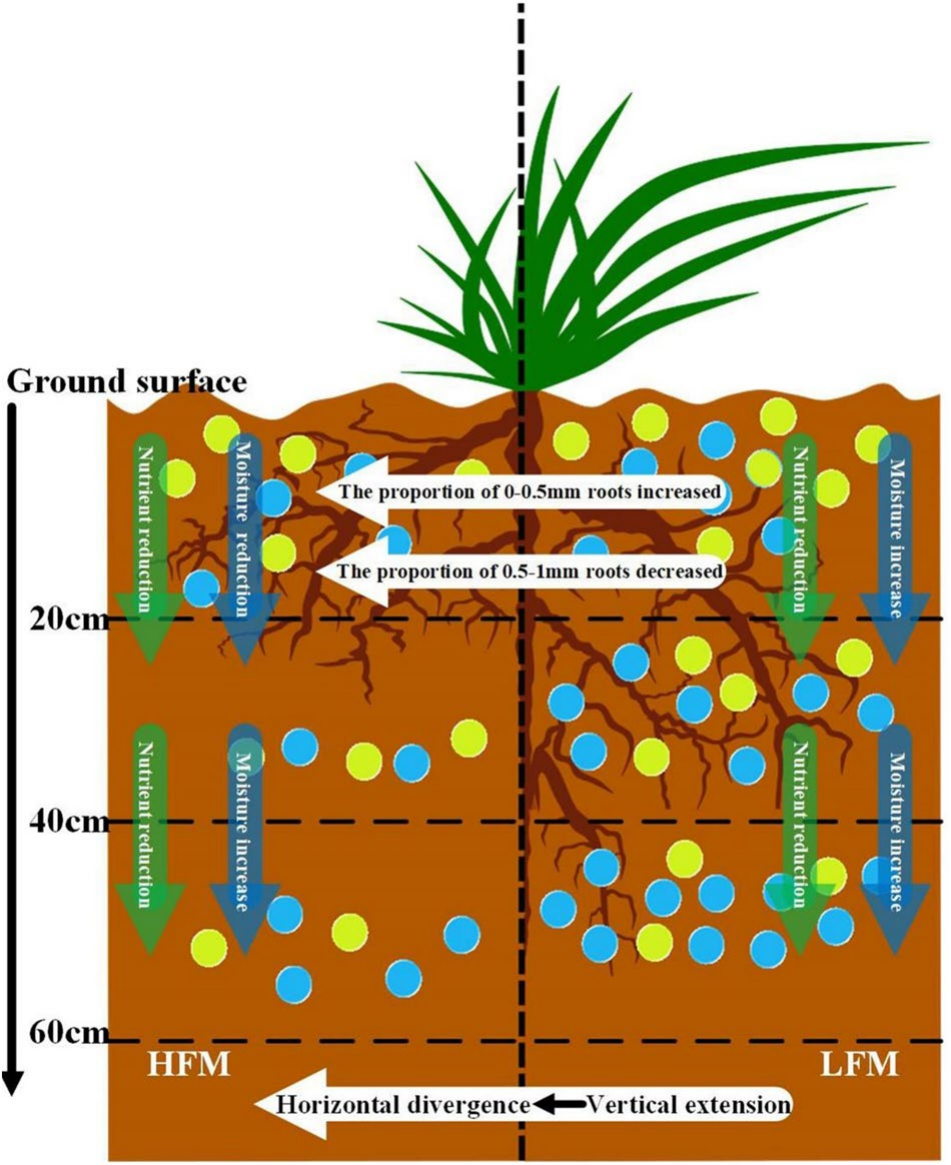
The influence mechanism of FTC on alpine meadow roots in the Nagqu River Basin,the blue circle represents moisture, and the yellow circle represents nutrients.The figure is generated with use of Inkscape0.92.3(https://inkscape.org/).

The above discussion shows that the damage of FTC to alpine meadows cannot determine the root distribution, the content and distribution of water and nutrients are the main factors affecting the distribution of alpine meadow roots, which is consistent with the hypothesis of this study. Therefore, the effect mechanism of FTCF on the distribution of alpine meadow roots is shown in Fig. 8.

As shown in Fig. 8, the effect of FTC on soil moisture and nutrients is the main reason for the two distribution modes of alpine meadow roots. In LFM, the soil moisture gradually increases from the surface to bottom (0–60 cm soil), the alpine meadow roots in LFM is distributed in 0–40 cm soil, and the growth mode is vertical extension. This growth mode can help alpine meadow roots absorb more water. With the increase in FTCF, the distribution of soil moisture is changing. In HFM, soil moisture in the surface (0–20 cm) is higher than that in the middle (20–40 cm). In order to absorb more water, the growth mode of alpine meadow roots changed from vertical extension to horizontal divergence, and the distribution range of roots is reduced from 0–40 to 0–20 cm. It is a better choice to reduce the root distribution to 0–20 cm soil than to extend roots to 40–60 cm soil. Therefore, the distribution of soil moisture is the main factor affecting the growth direction of roots, which fully shows the high openness of roots.

Meanwhile, in order to adapt to the environment changes, alpine meadow roots need to absorb a lot of water and nutrients. However, with the increase in FTCF, which increases the destruction of soil structure, changes the water holding and water conducting characteristics of soil, and accelerates the loss process of soil nutrients28, resulting in significant reduction in soil moisture and nutrient. At this time, the alpine meadow itself responded by increasing the content and specific gravity of 0–0.5 mm roots, and then increased the root surface area41 and enhanced the ability to absorb water and nutrients in the soil.

## Conclusions

In conclusion, the damage of FTC to alpine meadows has no significantly effect on the root distribution, and the change of soil moisture and nutrients by FTC is the main reason for the distribution of alpine meadow roots. The increase of FTCF can increase the loss of nutrients and weaken the ability of soil to retain water, resulting in the transfer of root distribution from 0–40 cm to 0–20 cm, and the increase in the proportion of 0–0.5 mm fine roots. In addition, the distribution range of alpine meadow roots is mainly affected by soil moisture, while the type and density of alpine meadow roots are jointly affected by soil moisture and nutrients.

If these changes are irreversible, as the global climate continues to warm, in the Nagqu River Basin will the moisture and nutrient content of 0–60 cm-depth soil continue to decrease? Will the distribution of water and nutrients continue to change? Will the roots of the alpine meadow gradually be distributed in the top soil? Will the properties of 0–60 cm soil be subverted? These issues need to be paid attention to and studied urgently. In the future, more attention should be paid to the monitoring of soil properties and alpine meadows.

## Data Availability

We declare that all the date in this study were available.
